# Thermal and physical impact of viscoplastic nanoparticles in a complex divergent channel due to peristalsis phenomenon: Heat generation and multiple slip effects

**DOI:** 10.1016/j.heliyon.2023.e17644

**Published:** 2023-07-05

**Authors:** Walid Aich, Khurram Javid, El Sayed Mohamed Tag-ElDin, Kaouther Ghachem, Irfan Ullah, Muhammad Asad Iqbal, Sami Ullah Khan, Lioua Kolsi

**Affiliations:** a Department of Mechanical Engineering , College of Engineering, University of Ha'il, Ha'il City, Saudi Arabia; b Department of Mathematics, Northern University, Wattar-Walli Road, Nowshera, 24110, KPK, Pakistan; c Faculty of Engineering and Technology, Future University in Egypt, New Cairo 11835, Egypt; d Department of Industrial Engineering and Systems, College of Engineering, Princess Nourah bint Abdulrahman University, P.O. Box 84428, Riyadh 11671, Saudi Arabia; e Department of Mathematics, University of Poonch, Rawalakot, AJK, Pakistan; f Department of Mathematics, Namal University, Mianwali 42250, Pakistan

**Keywords:** Viscoplastic nanofluid fluid, Complex peristaltic waves, Darcy's number, Second-order velocity slip, Thermal and concentration slips

## Abstract

In the advance studies, researchers have performed productive research contributions in the field of nanofluid mechanics under various biological assumptions. These contributions are fruitful to understand the applications of nanofluids in the various fields such as hybrid-powered engine, heart-diagnose, to prevent numerous diseases, heat exchanger, pharmaceutical processes, etc. The current analysis explores the combined effects of heat generation and chemical reaction on the peristaltic flow of viscoplastic nanofluid through a non-uniform (divergent) channel. The physical effects of second-order velocity slip, thermal slip and mass slip parameters on the rheological characteristics are also considered. To describe non-Newtonian effects, the Casson fluid is deployed. The greater wavelength assumption and low Reynolds number theory are used to attain the rheological equations. Numerical solutions of these governing equations associated with suitable boundary conditions are obtained via Mathematica symbolic software. The velocity magnitude of Casson fluid is higher than associated with Newtonian fluid. Radiation parameter has a vigorous impact in the reduction (enhancement) of temperature (mass concentration) profile. The porous parameter has a remarkable impact in reduction of temperature and velocity profile. Thermal enhancement is perceived by intensifying the chemical reaction parameter, and opposite inclination is noticed in mass concentration. Temperature has been demonstrated to be increased by increasing the Darcy number. The magnitudes of both axial velocity and temperature distribution are smaller in the presence of second-order velocity slip parameters effect as compared with no-slip condition. The magnitudes of axial velocity and mass (or, nanoparticle) concentration are augmented by accumulating the Prandtl number. A rise in Brownian parameter is noticed to depress the mass concentration. The present study has been used in bio-mechanical processes, nanomaterial devices, heat transfer enhancement, radiators, and electronics cooling systems.

## 1 Introduction

In bio-engineering domains, peristaltic (biomimetic) is an inherent phenomenon of the fluid motion through an elastic and flexible paths/channels/tubes due to contraction or expansion of successive propagating wave from a region of lower to higher pressure without using an external source. It is one of the significant functions for fluid motion in physiology and numerous industrial domains. In the human body, this pumping phenomena are beneficial in chime motion in the gastrointestinal tract, urine transport from kidney to bladder, food swallowing in the esophagus, in the vasomotion of small blood vessels. Additionally, lots of bio-industrial devices and operators are assembled by using the peristaltic code [[1], [2], [3]]. Jaffrin and Shapiro [4] studied the peristaltic flow of biological (seminal) liquid. Their mathematical analysis relies on lubrication and long-wavelength statements. Their analysis bestowed the theoretical foundation for various scrutiny, involving reflux, retrograde, trapping, and more phenomena. The mathematical formulations of biomimetic transportation of numerous Newtonian and non- Newtonian liquids have gained significant attention from scientists and mathematicians [[8], [9], [10], [11], [12], [13], [14], [15]]. All these studies were performed under biological approximations used by Jaffrin and Shapiro [4] in their flow analysis. Ali et al. [5] studies the long wavelength flow of a viscous fluid in a curved channel. They perceived the curvature effects on peristalsis features. Additionally, they have discussed the comparison among straight channel and curved channel on the flow of viscous liquid via peristalsis. They perceived that the curved channel has a lesser length of peristaltic rise as associated with straight channel. Javid et al. [6] analyzed the peristaltic motion of viscoelastic fluid in a curved regime under lubrication assumption. They observed that the viscoelastic parameter (at greater Weissenberg number) is dominant over the curvature effects. The biomimetic motion of non-viscous liquid (Couple stress fluid model) in a complex wavy domain is studied by Javid et al. [7] under porosity influences. They remarked that the porous medium has a significant effect in enhancement of pumping phenomena under lubrication approach. Asghar et al. [8] performed a theoretical analysis of complex peristaltic pumping of Ellis fluid in a non-uniform geometry under electro-osmotic impacts. They noticed enhancement in the peristaltic pumping under the larger strength of Ellis parameters. The electro-osmotic velocity reduces the velocity magnitude. Ahmed and Ali [9] performed a flow analysis of biological liquid in a porous heated channel under Joule heating influence. They noticed that sharp changes occurred in the velocity function because of the presence of the porous medium. Additionally, the magnetic and porous parameter has a dynamic role to control (decrease) the temperature profile. Narla and Tripathi [10] studied the blood flow in curved mircovessel under the influence of electric double layer. They noticed a reduction in the magnitude of the pressure gradient and wall shear stress by increasing curvature effects. Hina et al. [11] explored the influence of heat on peristaltic transportation of Powell-Eyring liquid (blood) in a curved channel with complaint walls. They have seen acceleration in axial velocity by increasing slip parameters. The viscous dissipation parameter has an astonishing role in an increment of temperature rises and heat flux. The physical influence of the Hall-device on the peristalsis of non-viscous fluid in an inclined asymmetric channel is studied by Hayat et al. [12] under low Reynolds number assumption. The velocity magnitude is enhanced by accumulating Hall parameter, Froude number and the angle of the magnetic field. They noticed enhancement in the temperature distribution by improving Froude number and Hall parameter.

In 1974, the Japanese researcher Taniguchi was firstly introduced the word “nanotechnology”. New inventions were started between 1980s and 1990s in the field of nanofluids because of their thermal enhancement feature of fluids. Nanofluids have dynamic applications in the various fields, such as fuel cells, cancer therapy, spray coating of materials of space technologies, micro-electronics, pharmaceutical suspensions, drug delivery systems, rocket propulsion, thermal insulation, hybrid cars, etc. All these mentioned potential applications of nanofluid are mostly owing to the improved thermal conductivity and Brownian motion dynamics which can be exploited to massive advantage. Choi and Eastman [13], firstly used the word nanofluid and determined that the thermal properties of ordinary liquids can be enhanced because of nanoparticle concentration. To start with, Buongiorno [14] deliberated the convective rheology of nanoliquids, and saw the thermal enhancement because of the centralization of nano-size solid particles. Aliabadi et al. [15] examined the experimental study related to heat transfer characteristics of liquid by using various nanoparticles. In their experimental research they noticed, thermal enhancement of Cu-water nanofluid is moderately sophisticated than Fe-water and Ag-water nanofluid. Another important experimental study related to concentration of Copper nanoparticles into water-based nanofluid. They perceived enhancement in the thermal efficiency of nanofluid by adding nanoparticles in the ordinary liquid. Some productive research of nanofluids can be seen in Refs. [[16], [17], [18], [19]]. Biomimetic rheology of nanoliquid through a curved geometry is deliberated by Hayat et al. [20]. They observed the effects of slip parameter, Ohmic heating and Hall parameter on the flow features under lubrication assumption. In saw that the nanofluid increases (reduces) the heat transfer rate (the temperature of fluid). While opposite behavior is perceived in the pattern of liquid motion and heat transfer features by increasing the magnetic and Hall parameter. Nadeem and Shahzadi [21] described the theoretical study for peristaltic pumping of nanoliquid in a curved domain under creeping phenomena. They have used two-phase nanofluid model in their flow anaylsis and used the concentration of three distinct nature of nanoparticles like $Cu,TiO_{2}\;\&\;Al_{2}O_{3}$ into water based. The curved channel has a smaller temperature profile as linked with straight channel. The outcomes of planar channel are obtained from higher numeric values of curvature parameter. Narla et al. [22] studied the effects of the electro-osmotic force on the peristaltic flow of nanoliquid in a curved pump to develop the proficiency of pump. The magnitude of temperature profile (nanoparticle concentration values) is augmented (reduced) by increasing Brinkman number and positive Joule heating parameter. Curvature parameter has a productive role in the reduction of entropy generation rate. Hina et al. [23] investigated the transportation of nanoliquid via sinusoidal waves through a curved path under creeping postulate. They acquired numerical solutions of flow features by using a shooting method. Nisar et al. [24] reported the peristaltic phenomenon for nanofluid with Carreau–Yasuda model. Das et al. [25] explored the thermal characteristics of hybrid nanofluid in endoscopic conduit.

In chemical and surgical domains, the collective influence of heat and mass transfer and chemical reaction have energetic roles. The chemical reaction can be of any order, however the most modest of which is the chemical reaction of the 1st order. In 1st order chemical reaction, the mass-concentration and the reaction rate are directly related to each other. In numerous chemical process, the chemical reaction among the mass concentration and working fluid takes place because of peristaltic pumping. Reddy and Kattamreddy [25] executed mathematical research associated with the influence of velocity slip and Joule heating on magneto-peristaltic transportation with chemical reaction. The physical impacts of the magnetic environment on the sinusoidal rheology of blood in narrow arteries is investigated by Vaidya et al. [26]. They performed the mathematical formulation under experimental verified physiological assumptions. They also detected the magnetic effect on numerous flow topographies with multiple chemical reactions. The larger magnetic force reduces the velocity and temperature profile. Shekar et al. [27] addressed the flow of viscoelastic nanoliquid (Jeffrey fluid model) in an asymmetric channel. They found the impact of MHD with chemical reaction on the peristaltic flow features under creeping theory. They noticed enhancement (increment) in the temperature and nanoparticle concentration profile by rising the chemical reaction (heat source parameter).

Motivated by the above discussion and broader engineering and medical applications, we propose of current continuation is to discuss the peristaltic rheology of Casson nanoliquid in a divergent (non-uniform) channel. This study is basically an extension of Shekar et al. [27]. In the present formulation, the viscoplastic fluid model is used instead of the viscoelastic fluid model. Furthermore, the investigation is performed in view of second-order slip effects. Also, the transportation phenomenon is influenced by thermal and mass slips constraints. Moreover, the chemical reaction and thermal radiation impact is considered. While the study of Shekar et al. [27] is performed without velocity and thermal slips effect. This is novelty of current formulation. This study is performed under the postulates of lubrication theory and greater wavelength. The rheological equations are transferred to a system of ODE's by using dimensionless variables and numerical solutions are acquired through the NDSolve method. Mathematica software 11.0 is used to acquire the numerical solutions of rheological equations under the creeping phenomena.

## 2 Mathematical formulation

**Fig. 1:**
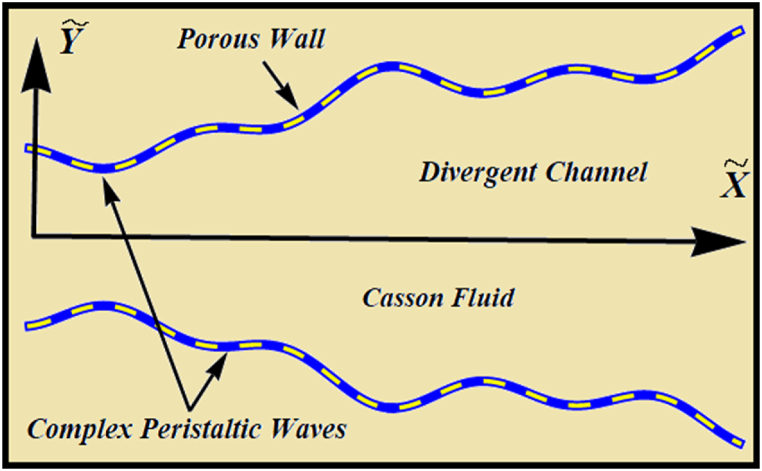
Flow diagram of non-uniform complex channel.

We consider the Casson nanofluid flow in a 2D divergent micro-channel under porosity effects with chemical reaction. We have considered Cartesian coordinate $\left(\tilde{X},\tilde{Y}\right)$ in the current formulation. Here, $\tilde{X}$ represents the wave-direction and $\tilde{Y}$ perpendicular to the $\tilde{X}-axis$. The flow is caused by an inherent train of peristaltic propulsion in the fixed frame of reference. Let, $\tilde{V}=\left(\tilde{W},\tilde{U},0\right)$ be the velocity-vector for the current flow problem. Here, $\tilde{W}$ represents the velocity component in axial direction and $\tilde{U}$ denotes the velocity component in orthogonal to axial direction. The diagram of flow problem under consideration is shown in Fig. 1. The complex pattern of upper $\left(\tilde{H}\right)$ and lower $\left(-\tilde{H}\right)$ walls of the channel are mathematically defined as [28]:

(1) $$\tilde{H}=A+\left(\tilde{X}-\Omega \tilde{t}\right)\tan\;\varphi +\sum_{i=1}^{3}c_{i}\;\mathrm{Cos}\left(\propto_{i}\frac{\pi }{\sigma }\left(\tilde{X}-\Omega \tilde{t}\right)\right),\left(Upper\;Wall\right)$$

(2) $$-\tilde{H}=-A-\left(\tilde{X}-\Omega \tilde{t}\right)\tan\;\varphi -\sum_{i=1}^{3}c_{i}\;\mathrm{Cos}\left(\propto_{i}\frac{\pi }{\sigma }\left(\tilde{X}-\Omega \tilde{t}\right)\right),\left(Lower\;Wall\right)$$

In Eqs. (1), (2), A is half channel-width, φ is non-uniform (divergent) parameter, Ω is wave speed, $c_{i}\;\left(i=1-3\right)$ are distinct amplitudes of peristaltic wave, σ is wavelength and $\propto_{i}\;\left(i=1-3\right)$ are distinct flow parameters related to peristaltic wave [28].

In the current era, lots of research has been performed related to the rheology of unsteady non-Newtonian fluids via numerous regimes possessing a definite yield value due to their usages in biological technologies and polymer processing engineering. Casson fluid is one of the most well-known liquids. The Casson model is basically a structure-based model used to describe the rheology of viscoplastic liquid. For an incompressible and isotropic rheology, the flow equation of a non-Newtonian Casson liquid is [[29], [30], [31], [32]].

(3) $$\sigma_{nm}=\left\{ \begin{array}{c} 2\left(\mu_{B}+\frac{Py}{\sqrt{2\pi }}\right)\varepsilon_{nm},\pi \geq \pi_{0}\\ 2\left(\mu_{B}+\frac{Py}{\sqrt{2\pi_{0}}}\right)\varepsilon_{nm},\pi <\pi_{0.} \end{array}\right.$$

The physical quantities appeared in Eq. (3) are Py which signifies the yield stress of fluid, $\sigma_{nm}$ denotes the (n,m)−th stress tensor component, $\pi =\varepsilon_{nm}\varepsilon_{mn}$ and $\varepsilon_{nm}$ represents the (n,m)−th deformation rate component, $\pi_{0}$ demonstrates the critical value of π based on the non-Newtonian model, $\mu_{B}$ symbolizes plastic dynamic viscosity of non-Newtonian fluid. At Py=0, the stress tensor component of viscous fluid is recovered. This model is also known as biviscosity fluid model. In the medical domain, this model is mostly used for the mathematical formulation of blood rheology in small vessels and arteries. It is used under the circumstances at less shear rate. Examples of Casson fluid include concentrated fruit juice, chocolate melts, honey, jelly, soup, paints, and sauce. Furthermore, this model can be used in creating mathematical models for hemodialyzer's and blood oxygenators.

The governing equations associated to the two-dimensional flow of an incompressible Casson nanofluid in term of Cartesian coordinates are given by Refs. [[29], [30], [31], [32]]:

(4) $$\frac{\partial \tilde{W}}{\partial \tilde{X}}+\frac{\partial \tilde{U}}{\partial \tilde{Y}}=0\text{,}$$

$$\frac{\partial \tilde{W}}{\partial \tilde{t}}+\tilde{W}\frac{\partial \tilde{W}}{\partial \tilde{X}}+\tilde{U}\frac{\partial \tilde{W}}{\partial \tilde{Y}}=-\frac{1}{\varphi_{f}}\frac{\partial \tilde{P}}{\partial \tilde{X}}+\frac{1}{\varphi_{f}}\left(\frac{\partial {\tilde{\tau }}_{\tilde{X}\tilde{X}}}{\partial \tilde{X}}+\frac{\partial {\tilde{\tau }}_{\tilde{X}\tilde{Y}}}{\partial \tilde{Y}}\right)-\frac{\mu }{K^{*}\varphi_{f}}+\left(1-\Theta_{0}\right)G\varsigma \left(\tilde{\varphi }-\varphi_{0}\right)$$

(5) $$+\left(\frac{\varphi_{p}-\varphi_{f}}{\varphi_{f}}\right)G\varsigma^{\prime }\left(\tilde{\Theta }-\Theta_{0}\right)\text{,}$$

(6) $$\frac{\partial \tilde{U}}{\partial \tilde{t}}+\tilde{W}\frac{\partial \tilde{U}}{\partial \tilde{X}}+\tilde{U}\frac{\partial \tilde{U}}{\partial \tilde{Y}}=-\frac{1}{\varphi_{f}}\frac{\partial \tilde{P}}{\partial \tilde{Y}}+\frac{1}{\varphi_{f}}\left(\frac{\partial {\tilde{\tau }}_{\tilde{X}\tilde{Y}}}{\partial \tilde{X}}+\frac{\partial {\tilde{\tau }}_{\tilde{Y}\tilde{Y}}}{\partial \tilde{Y}}\right)-\frac{\mu }{K^{*}\varphi_{f}}\text{,}$$

$$\frac{\partial \tilde{\varphi }}{\partial \tilde{t}}+\tilde{W}\frac{\partial \tilde{\varphi }}{\partial \tilde{X}}+\tilde{U}\frac{\partial \tilde{\varphi }}{\partial \tilde{Y}}=\gamma \left(\frac{\partial^{2}\tilde{\varphi }}{\partial {\tilde{X}}^{2}}+\frac{\partial^{2}\tilde{\varphi }}{\partial {\tilde{Y}}^{2}}\right)+\frac{1}{\varphi_{f}C_{f}}\left({\tilde{\tau }}_{\tilde{X}\tilde{X}}\frac{\partial \tilde{W}}{\partial \tilde{X}}+{\tilde{\tau }}_{\tilde{X}\tilde{Y}}\left(\frac{\partial \tilde{W}}{\partial \tilde{Y}}+\frac{\partial \tilde{U}}{\partial \tilde{X}}\right)+{\tilde{\tau }}_{\tilde{Y}\tilde{Y}}\frac{\partial \tilde{U}}{\partial \tilde{Y}}\right)-\frac{1}{\varphi_{f}C_{f}}\frac{\partial q_{r}}{\partial \tilde{Y}}+$$

(7) $$\zeta \left(d_{b}\left(\frac{\partial \tilde{\Theta }}{\partial \tilde{X}}\frac{\partial \tilde{\varphi }}{\partial \tilde{X}}+\frac{\partial \tilde{\Theta }}{\partial \tilde{Y}}\frac{\partial \tilde{\varphi }}{\partial \tilde{Y}}\right)+\frac{d_{T}}{T_{0}}\left({\left(\frac{\partial \tilde{\varphi }}{\partial \tilde{X}}\right)}^{2}+{\left(\frac{\partial \tilde{\varphi }}{\partial \tilde{Y}}\right)}^{2}\right)\right)+Q_{0}\text{,}$$

(8) $$\frac{\partial \tilde{\Theta }}{\partial \tilde{t}}+\tilde{W}\frac{\partial \tilde{\Theta }}{\partial \tilde{X}}+\tilde{U}\frac{\partial \tilde{\Theta }}{\partial \tilde{Y}}=d_{b}\left(\frac{\partial^{2}\tilde{\Theta }}{\partial {\tilde{X}}^{2}}+\frac{\partial^{2}\tilde{\Theta }}{\partial {\tilde{Y}}^{2}}\right)+\frac{d_{t}}{\varphi_{0}}\left(\frac{\partial^{2}\tilde{\varphi }}{\partial {\tilde{X}}^{2}}+\frac{\partial^{2}\tilde{\varphi }}{\partial {\tilde{Y}}^{2}}\right)-K_{1}\left(\tilde{\Theta }-\Theta_{0}\right)\text{.}$$

Here, $\tilde{P}$ is pressure, $\tilde{\tau }$ is Cauchy stress tensor, ${\tilde{\tau }}_{\tilde{X}\tilde{X}},{\tilde{\tau }}_{\tilde{X}\tilde{Y}}\&{\tilde{\tau }}_{\tilde{Y}\tilde{Y}}$ are components of extra stress tensor, μ is dynamic viscosity, $\tilde{\varphi }$ is temperature profile, $\tilde{\Theta }$ is the nanoparticle phenomena, ambient values of $\tilde{\varphi }$ and $\tilde{\Theta }$ is $\varphi_{0}$ and $\Theta_{0}$, $\varphi_{f}$ is fluid density, $\varphi_{p}$ is particle density, C is the volumetric expansion coefficient, $\zeta ={\left(\varphi C\right)}_{p}/{\left(\varphi C\right)}_{f}$ is ratio effective heat capacity of nanoparticles to effective heat capacity of fluid, $d_{b}$ is Brownian diffusion coefficient, $d_{T}$ is thermophoretic diffusion coefficient, ς is thermal expansion coefficient, $C_{p}$ is volumetric expansion coefficient of nanoparticles, $\varsigma^{\prime }$ is expansion with concentration coefficient, G is gravitational field, $K^{*}$ is permittivity of boundary walls, $K_{1}$ is chemical reaction parameter and $Q_{0}$ is heat source/sink parameter.

The mathematical illustration of radiative heat flux is demarcated via Eq. (9) as [33]:

(9) $$Q_{r}=-\frac{4\varrho }{3k}\frac{\partial {\tilde{\varphi }}^{4}}{\partial \tilde{Y}}\text{,}$$

where, ϱ is Stefan-Boltzmann constant and k is mean absorption coefficient.

The fixed (unsteady state) and wave (steady state) frame transformation is given by [30].

(10) $$\tilde{x}=\tilde{X}-\Omega \tilde{t},\tilde{y}=\tilde{Y},\tilde{P}\left(\tilde{x}\right)=\tilde{P}\left(\tilde{X},\tilde{t}\right),\tilde{u}=\tilde{U},\tilde{w}=\tilde{W}-\Omega \text{.}$$

Introducing the following scaling variables:

$x=\tilde{x}/\sigma$ is axial component, $y=\tilde{y}/\sigma$ is transverse coordinate, $w=\tilde{w}/\Omega$ is axial velocity, $u=\tilde{u}/\Omega$ is transverse velocity, $t=\Omega \tilde{t}/\sigma$ is time, $P=a^{2}\tilde{P}/\Omega \sigma \mu$ is pressure, $\varphi =\left(\tilde{\varphi }-\varphi_{0}\right)/\left(\varphi_{1}-\varphi_{0}\right)$ is temperature profile, $\Theta =\left(\tilde{\Theta }-\Theta_{0}\right)/\left(\Theta_{1}-\Theta_{0}\right)$ is nanoparticle phenomena, $Gr={\left(1-\Theta_{0}\right)\varphi }_{f}G\varsigma A^{2}\left(\tilde{\varphi }-\varphi_{0}\right)/\Omega \mu$ is Grashof number, $Gm=\left(\varphi_{p}-\varphi_{f}\right)G\varsigma {\prime A}^{2}\left(\varphi_{1}-\varphi_{0}\right)/\Omega \mu_{nf}$ is local nano particle Grashof number, $nb=\zeta d_{b}\left(\Theta_{1}-\Theta_{0}\right)/\vartheta$ is Brownian motion, $nt=\zeta d_{t}\left(\varphi_{1}-\varphi_{0}\right)/\Theta_{0}\vartheta$ is thermophoresis, $a_{i}=c_{i}/A\;\left(i=1-3\right)$ are different amplitudes, $R_{e}=\Omega A/\vartheta$ is Reynolds number, δ=A/σ is the wave number, $h=\tilde{H}/A$ is upper wall, $-h=-\tilde{H}/A$ is lower wall, $Rn=16\varrho \varphi_{0}/3k\mu C_{f}$ is Brownian motion, $\mathrm{Pr}=\mu C_{f}/\gamma$ is Prandtl number, $\gamma =Q_{0}A^{2}/\gamma \left(\varphi_{1}-\varphi_{0}\right)$ is the heat source/sink parameter, $Ec=\Omega^{2}/{\left(\varphi_{1}-\varphi_{0}\right)C}_{f}$ is Eckert number, $\xi =K_{1}A^{2}/d_{b}$ is chemical reaction, $Da=A/\sqrt{K^{*}}$ is Darcy's number (porosity parameter), Br=EcPr is Brinkman number, and $\tau_{ij}=\left(A/\Omega \mu \right){\tilde{\tau }}_{\tilde{i}\tilde{j}}$ are extra-stress tensor components.

The velocity field in term of stream function is defined as:

(11) $$w=\psi_{y},u=-\delta \psi_{x}\text{.}$$

Here, we will perform some mathematical formulations. Firstly, we will shift the flow system (Eqs (4)–(8)) from unsteady to steady-state by using Eq. (10). Secondly, we will make this rheological system (steady state) into dimensionless form by using scaling variables. In the third step, we will rewrite the rheological system in to stream function by using Eq. (11). Here, we get a system of partial differential equations (PDE's). In fourth step, we will apply two effective physiological assumptions: one is the creeping theory $\left(R_{e}\to 0\right)$ and second is the larger wavelength (σ→∞). Mathematically, in the creeping phenomena, the inertial force is considered very small as associated with the viscous force. While in the long wavelength postulates, the wavelength among two consecutive crusts (or, troughs) is much larger via compared with the half width of the flow regime. After using these two biological assumptions, the system of PDE's reduce to ODE's. The simplified form of flow system is expressed as:

(12) $$-\frac{\partial P}{\partial x}+\frac{\partial \tau_{xy}}{\partial y}+Gr\varphi +Gm\Theta -\frac{1}{Da^{2}}\left(w+1\right)=0\text{,}$$

(13) $$\frac{\partial P}{\partial y}=0\text{,}$$

(14) $$\left(1+RnPr\right)\frac{\partial^{2}\varphi }{\partial y^{2}}+Br\tau_{xy}\frac{\partial w}{\partial y}+nbPr\frac{\partial \Theta }{\partial y}\frac{\partial \varphi }{\partial y}+ntPr{\left(\frac{\partial \varphi }{\partial y}\right)}^{2}+\beta =0\text{,}$$

(15) $$\frac{\partial^{2}\Theta }{\partial y^{2}}+\frac{Nt}{Nb}\frac{\partial^{2}\varphi }{\partial y^{2}}-\xi \Theta =0\text{.}$$

where, $\tau_{xy}=\left(1+\frac{1}{\alpha }\right)\frac{\partial w}{\partial y}$ and $w=\frac{\partial \psi }{\partial y}$. Here, $\alpha =\mu_{B}\sqrt{2\pi }/Py$ is known as viscoplastic parameter or the upper limit apparent viscosity coefficient. According to the convenient, some authors have used it in their study as a viscoelastic model and few of them have used this model in their flow analysis as a biviscosity model [[29], [30], [31], [32]].

Alternative form of above system of Eqs. (12), (13), (14), (15) in terms of stream function in the absence of pressure function.

(16) $$\left(1+\frac{1}{\alpha }\right)\frac{\partial^{4}\psi }{\partial y^{4}}+Gr\frac{\partial \varphi }{\partial y}+Gm\frac{\partial \Theta }{\partial y}-\frac{1}{Da^{2}}\frac{\partial^{2}\psi }{\partial y^{2}}=0\text{,}$$

(17) $$\left(1+RnPr\right)\frac{\partial^{2}\varphi }{\partial y^{2}}+Br\left(1+\frac{1}{\alpha }\right){\left(\frac{\partial^{2}\psi }{\partial y^{2}}\right)}^{2}+nbnr\frac{\partial \Theta }{\partial y}\frac{\partial \varphi }{\partial y}+ntPr{\left(\frac{\partial \varphi }{\partial y}\right)}^{2}+\beta =0\text{.}$$

For Eqs. (16), (17), the associate boundary conditions are [[31], [32], [33], [34]]:

$$\beta_{2}\left(1+\frac{1}{\alpha }\right)\frac{\partial^{3}\psi }{\partial y^{3}}+\beta_{1}\left(1+\frac{1}{\alpha }\right)\frac{\partial^{2}\psi }{\partial y^{2}}+\frac{\partial \psi }{\partial y}=-1,\varphi +\gamma_{1}\frac{\partial \varphi }{\partial y}=0,\Theta +\gamma_{2}\frac{\partial \Theta }{\partial y}=0,at\;y=h_{1}\text{,}$$

$${-\beta }_{2}\left(1+\frac{1}{\alpha }\right)\frac{\partial^{3}\psi }{\partial y^{3}}-\beta_{1}\left(1+\frac{1}{\alpha }\right)\frac{\partial^{2}\psi }{\partial y^{2}}+\frac{\partial \psi }{\partial y}=-1,\varphi -\gamma_{1}\frac{\partial \varphi }{\partial y}=0,\Theta -\gamma_{2}\frac{\partial \Theta }{\partial y}=0,at\;y=h_{2}\text{.}$$

Here, $\beta_{1}$ and $\beta_{2}$ are first and second-order slip parameters, $\gamma_{1}$ is thermal slip parameter and $\gamma_{2}$ is nanoparticle concentration slip parameter. In the medical domains, some time the temperature and nanoparticles concentration of nanofluids is enhanced or reduced due to variations of the flow parameters under lubrication theory. To control the physical behavior of the temperature and nanoparticles concentration up to the requirement of medical and engineering domains, the thermal slip and nanoparticle concentration slip conditions are used. The impacts of partial slip conditions are significant in improving the regulators of artificial heart. These have a strong influence on the blood transportation in peristaltic pumps. Additionally, to overcome the effects of heat and nanoparticle concentration on the boundary walls of flow geometry by using partial slip conditions are addressed by many authors [[33], [34], [35], [36], [37], [38], [39]]. Furthermore, which have massive applications to control the motion of nanofluid in nano-size channel or micro-tubes under the lubrication theory and larger wavelength postulate. The physical conditions and range of embedded parameters are given below in a Table 1:

**Table 1:** The physical conditions and range of embedded parameters.

Flow Variable	Range	Flow Variable	Range
α	[1,10]	Da	[0.1,2]
nb	[0.1,2]	Rn	[0.1,1]
nt	[0.1,8]	Pr	[1,5]
$\beta_{1},\beta_{2}$	[0,0.1]	ξ	[0,3]
β	[0,2]	$\gamma_{1},\gamma_{2}$	[0,0.3]
Br	[0,2]		

## 3 Solution methodology

In the current formulation's, these values are chosen in that criteria where the proper flow diagram of flow features are easily obtained. Additionally, if the flow diagram is plotted outside the mentioned domain, then the graphical behavior of flow features is strongly distrusted. In the current formulations, three physical conditions are used: one is creeping theory, second is larger wavelength and third is law of heat conduction. By using these conditions, the flow equations become linear ODE and easily to solve by using numerical technique in Mathematica software.

The NDSolve is a numerical technique and is mostly used to provide approximate solution of differential equation (DE). This is also known as a numerical DE solver. This method is applicable for both ODEs and PDEs. It is also suitable to solve the mixture of algebraic and differential equations. NDSolve finds the numerical solution of an interpolating function such as $u_{i}$. We find the solution of interpolating function $u_{i}$ over the given domain of independent variable, say t, starts from $t_{\min}$ to $t_{\max}$. This is basically an iterative technique that starts at a fixed point of independent variable, and then try to cover the given domain from $t_{\min}$ to $t_{\max}$. In the start of technique, the initial or boundary conditions for the dependent variables and their derivatives should be defined at particular points *t*. If the condition is defined only at one point, then it will be an IVP. While the BVP occurs more than one point. This solver is used for linear and higher order derivative problems. Whenever the conditions are applied in the NDSolve technique, it automatically finds the solutions of arbitrary constants associated with initial conditions that you have not found easily. This method has more accuracy and efficiency as compared with lots of other numerical techniques. Additionally, it is a built-in method in Mathematica and easily handled [40].

## 4 Validation of results

**Fig. 2:**
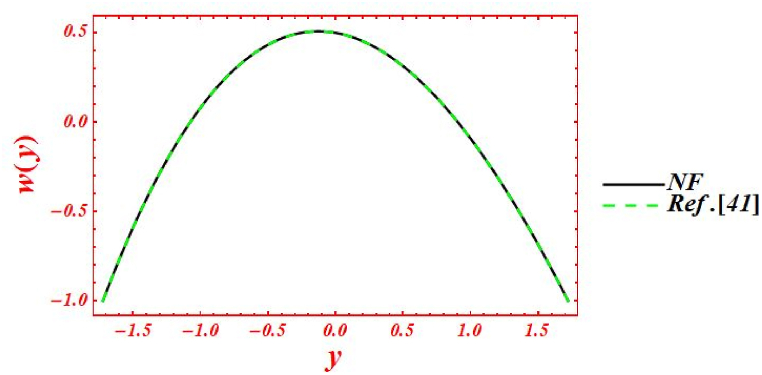
Velocity comparison between the Newtonian fluid (NF) and Ref. [41].

The results of Burn and Parkes [41] can easily be derived from the governing equations by ignoring contributions of following flow parameters. Mathematically, the outcomes of Burn and Parkes [41] are retrieved by substituting following restrictions in the rheological equations. Let, α→∞, $\beta_{1}=0,\beta_{2}=0,nb=0,\varphi =0,\gamma_{1}=0\;\&\;\gamma_{2}=0\text{.}$ The comparison between the current study and Ref. [41] is drawn in Fig. 2. Authors have performed their flow analysis in both symmetric channels and axially symmetric pipes under the suppositions of low Reynolds number. They have used Newtonian liquid in their formulation and motion is taken place due to sinusoidal contraction and relaxation of wave. These results clearly validate and guarantee the correctness and accuracy of current formulation and mathematical technique.

## 5 Results and discussions

The peristaltic rheology of an incompressible Casson nanofluid in a porous medium under the influences of second-order velocity, thermal and mass-concentration slips with chemical reaction has been studied in the present formulation. The brunt of flow parameters such as Brinkman number (Br), Local nano particle Grashoff number (Gm), Brownian motion (nb), Thermophoresis (nt), Prandtl number (Pr), Darcy's number (Da), Heat Source/Sink parameter (β), Chemical Reaction Parameter (ξ), velocity slips $\left(\beta_{1},\beta_{2}\right)$, thermal slip $\left(\gamma_{1}\right)$, concentration slip $\left(\gamma_{2}\right)$, viscoplastic parameter (α), radiation parameter (Rn) and Grashof number (Gr) on the axial velocity, temperature distribution and nanoparticle concentration are discussed by a set of graphs. The fixed values of parameters is defined as $\alpha =1,Gr=0.5,Gm=0.5,Da=1.5,nt=2,Rn=1,Br=2,\mathrm{Pr}=1,\xi =1,\beta =0.1,\beta_{1}=0.1,\beta_{2}=0.1,\gamma_{1}=0.1,\gamma_{2}=0.1,Q=2,d=1$.

**Fig. 3:**
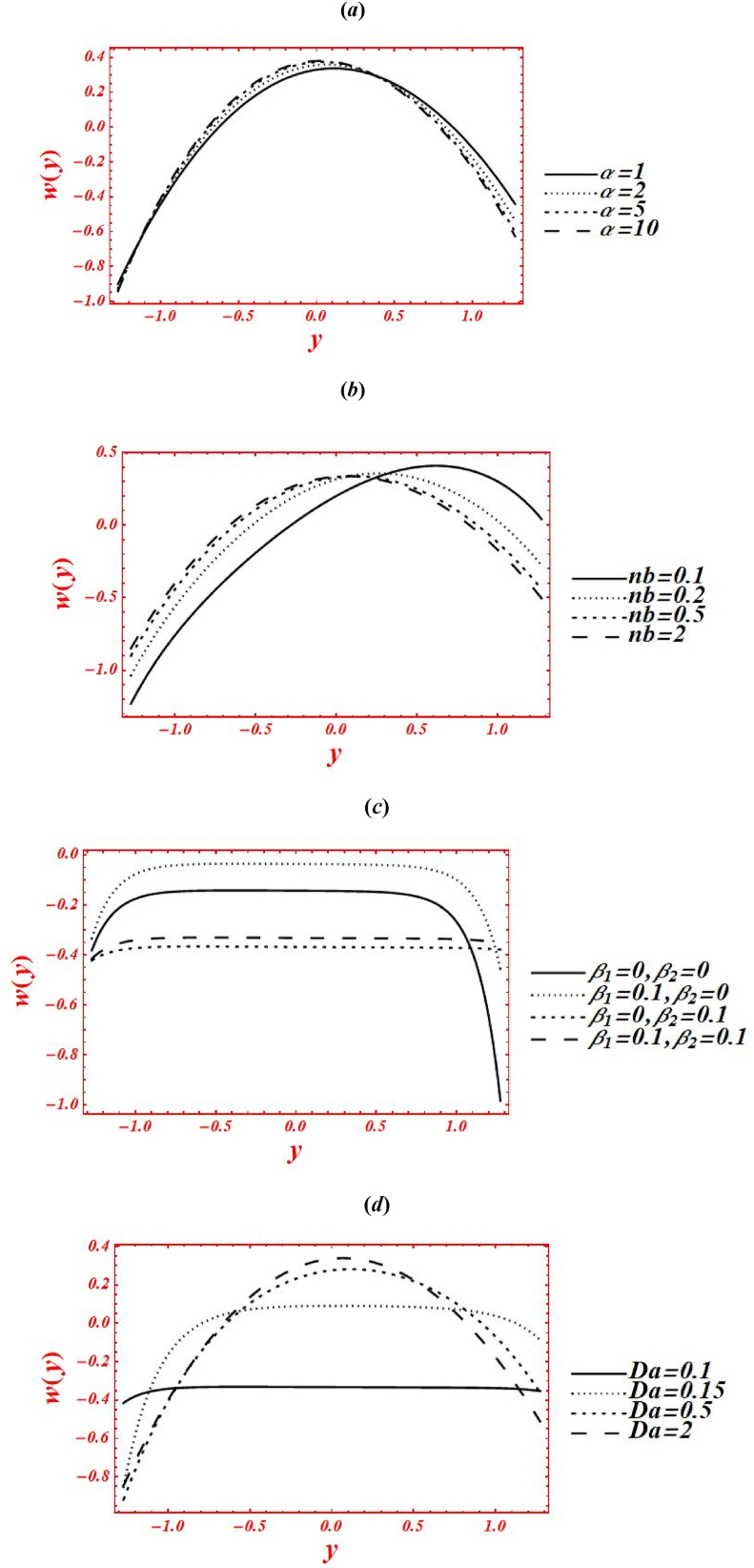

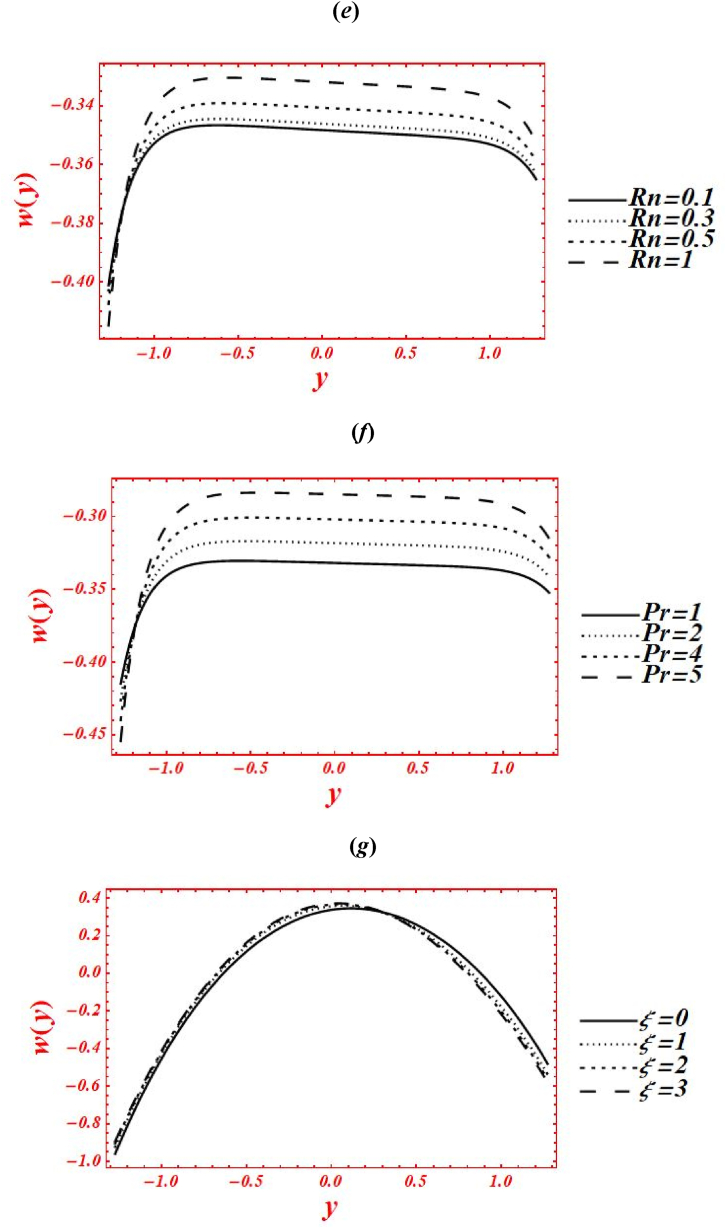
(a–g): (*a*) Velocity profile for Casson parameter (*b*) Velocity profile for Brownian motion (nb) (*c*) Velocity profile for second-order velocity slips $\left(\beta_{1},\beta_{2}\right)$, (*d*) Velocity profile for Darcy's number (Da), (*e*) Velocity profile for radiation parameter (Rn), (*f*) Velocity profile for Prandtl number (Pr), (*g*) Velocity profile for chemical reaction parameter (ξ).

Consequences of numerous rheological parameters on the axial velocity (w(y)) have been plotted are shown in Fig. 3(*a* –*g*). The asymmetric nature in all the graphs of velocity profile is observed due to non-uniform nature of flow geometry. The impacts of Casson parameter (α) on w(y) of nanofluid under second-order velocity slip and heat generation with chemical reaction is plotted in Fig. 3(*a*). Change the Casson (viscoplastic) parameter from 1 to 10 shows that asymmetry nature of velocity graph of nanofluid is disturbed and magnitude of w(y) shifted toward the center of flow geometry. Additionally, the velocity magnitude is also enhanced by increasing Casson parameter. Here, it is vital to noted that the result of a viscous fluid is acquired at larger strength of viscoplastic parameter, at α=10, under second-order velocity slip and heat generation with the chemical reaction. In the physical way, the viscoplastic effects over comes the viscous effects. The parabolic character of velocity is maintained in the smallest vigor of porosity effects at Da=1.5. The graph of w(y) is shifted to downward in the vicinity of the channel at yε[0.35,1.285], and moved toward the upward at yε[−1.285,0.35]. Fig. 3(*b*) deals with the effects of Brownian motion (nb) on w(y) of nanofluid under heat generation and second-order velocity slips. Here, it is shown that as nb is increase from 0.1 to 2, the graph of velocity profile is shifted from right half to center of the channel. Furthermore, the graph is shifted from upper to lower half of the regime at yε[0.35,1.285] near the boundary wall as rise the Brownian parameter. However, the magnitude of w(y) is reduced near the left boundary wall from −1.25 to −0.85 as nb increases. Graphically, the Brownian motion parameter control the collusion and random motion of nano-size particles with each other in the flow geometry which leads to generations of sufficient amount of heat and hence the temperature is an absolute sense. The outcomes of Brown motion parameter are obtained under fixed value of porosity at Da=1.5 and chemical reaction parameter at ξ=1. The impacts of second-order velocity slips $\left(\beta_{1},\beta_{2}\right)$ on w(y) is plotted in Fig. 3(*c*) under Brownian effects. Here, different nature in the behavior of velocity profile is seen. Maximum magnitude in w(y) is obtained at $\beta_{1}=0.1\;\&\;\beta_{2}=0$. While the smallest magnitude in w(y) is seen at $\beta_{1}=0\;\&\;\beta_{2}=0.1$. In physical point of view, the velocity slip parameters have remarkable act in reduction of w(y). The magnitude of velocity can be controlled by second-order velocity slip parameters under chemical reaction. All these graphs are shifted toward the lower half of channel under slip effects. Additionally, the abrupt varies (boundary layers, BL) are noticed in velocity graph in two cases: one is at $\beta_{1}=0\text{,}$ and $\beta_{2}=0.1$, and second one is at $\beta_{1}=0.1\text{,}$ and $\beta_{2}=0.1$. In the physical prospectives, the influence of slips at the boundary walls overcome the rheological resistance which help to boost the velocity at boundary. The influence of porosity parameter (Darcy's number (Da)) on w(y) of nanofluid is drawn in Fig. 3(*d*) under heat generation and second-order velocity slips. The magnitude of w(y) is enhanced as increases Da from 0.1 to 2. The sharp changes in velocity graph of Casson nanofluid is seen for smaller Da, say Da=0.1. The parabolic graph is obtained for larger values of Da. In physical point of view, parabolic nature of velocity graph of nanofluid is intensely involved in larger porosity effects. The effects of radiation parameter (Rn) and Prandtl number (Pr) on w(y) of nanofluid are drawn in Fig. 3(*e*) & **(*f*)** under second-order velocity slips and porosity effects. The elevation behavior in the velocity magnitude is observed in both figures by increasing Rn from 0 to 1 and Pr from 1 to 5. In Fig. 3(*e*), it is shown that the kin influence of conduction heat transfer overcomes thermal radiation transfer, and in the conclusion, the intermolecular movement of nanoparticles is boosted. Furthermore, it played a dynamic role in enhancement of velocity at the channel center. While in Fig. 3(*g*), as the numeric value of Prandtl number rises the physical effects of kinematic viscosity is enhanced in the velocity profile, and the influence of thermal diffusivity on the velocity is reduced. Fig. 3(*g*) shows the variation of w(y) for distinct values of chemical reaction parameter (ξ) under porosity and second-order slip effects. The asymmetric behavior is predicted in the graph of w(y) in the absence of chemical reaction parameter, say ξ=0. The graph of w(y) is shifted to the channel center by increasing ξ from 0 to 3. Additionally, it shows decreasing (increasing) behavior in w(y) at right (left) side of center, at y=0.

**Fig. 4:**
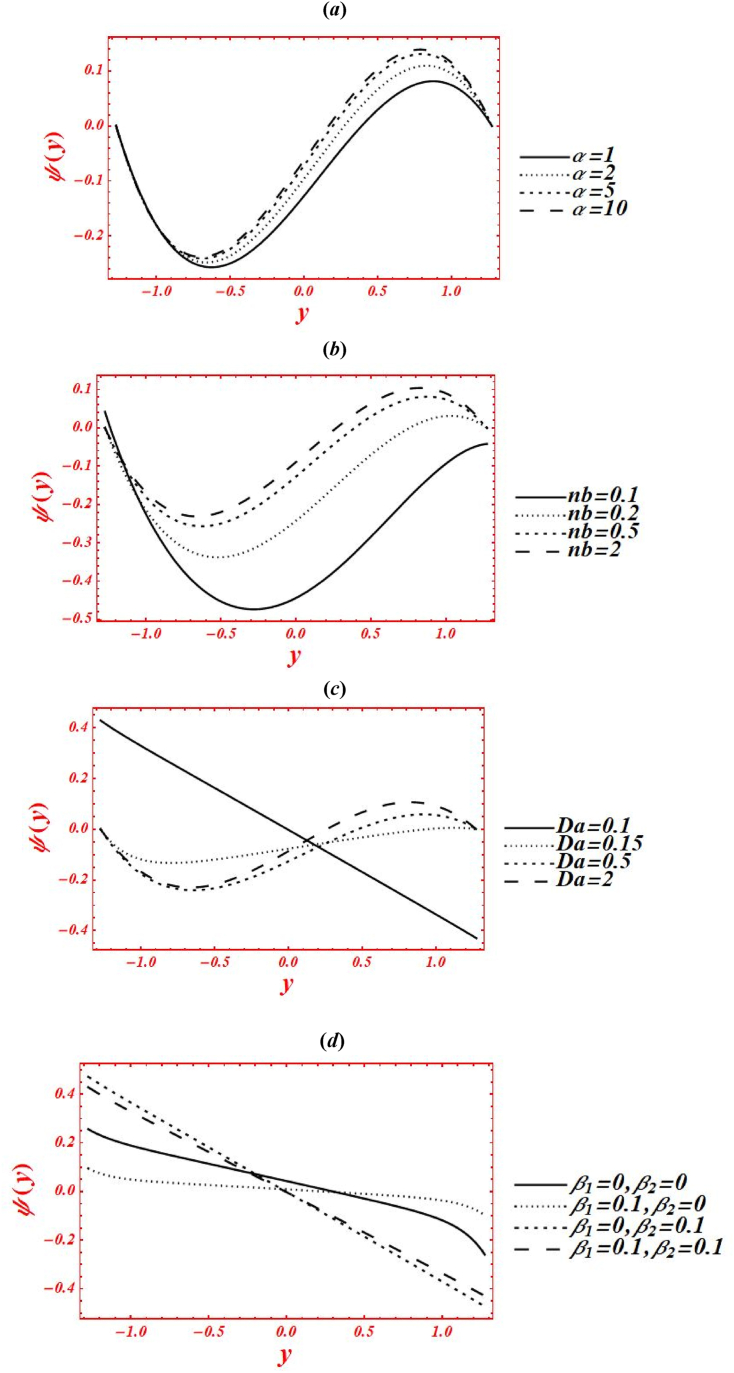

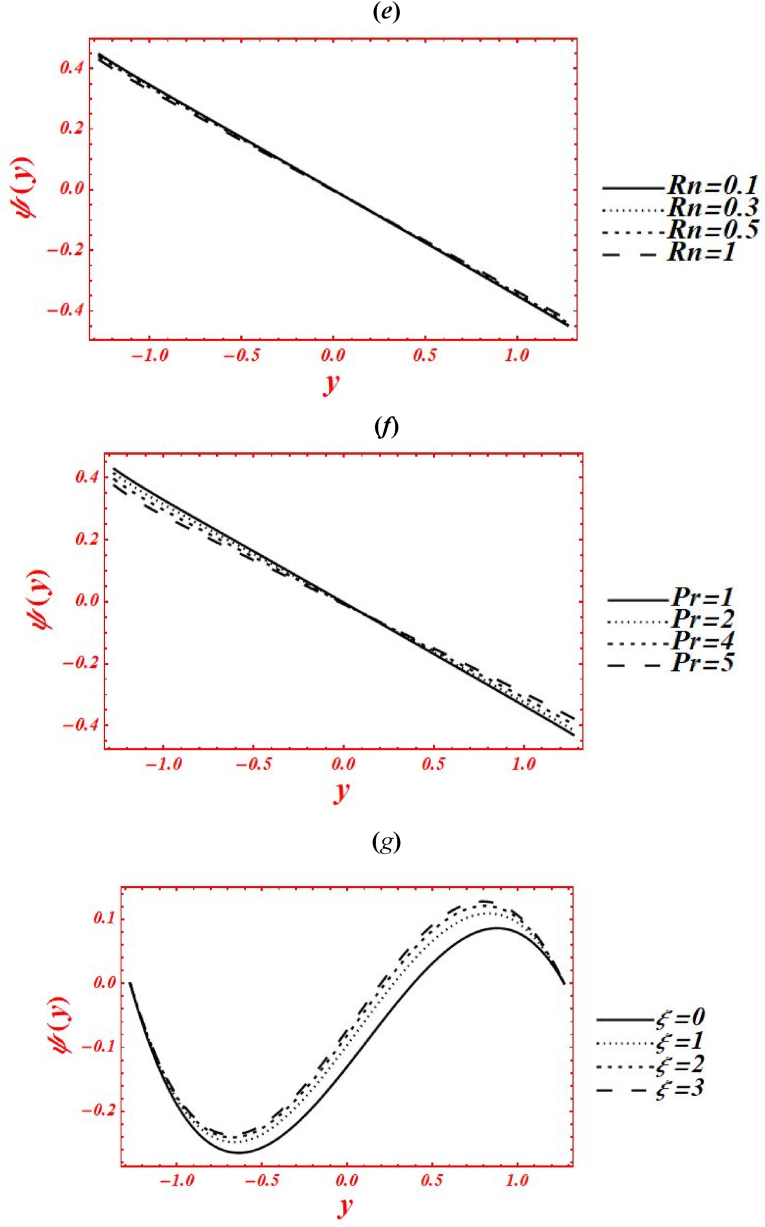
(a–g): (a) Stream function for Casson parameter (α), (*b*) Stream function for Brownian motion (nb), (*c*) Stream function for second-order velocity slips $\left(\beta_{1},\beta_{2}\right)$, (*d*) Stream function for Darcy's number (Da), (*e*) Stream function for radiation parameter (Rn), (*f*) Stream function for Prandtl number (Pr), (*g*) Stream function for chemical reaction parameter (ξ).

Effects of numerous flow parameters on the stream function (ψ(y)) have been plotted are shown in Fig. 4(*a* –g). A sinusoidal wave is produced in the graphs of stream function. The steep of stream function is upward and behaves like a crust in the region yε[0,1.286], and the slope of graph is downward and behaves like a trough in the region yε[−1.286,0]. The impact of Casson parameter (α) on ψ(y) of nanofluid under second-order velocity slip and heat generation with the chemical reaction is plotted in Fig. 4(*a*). Change the Casson (viscoplastic) parameter from 1 to 10 shows that the magnitude of ψ(y) is enhanced in upper-half of the channel and reduced in lower-half of the channel. Here, it is vital to noted that the graph of ψ(y) for a viscous fluid, say α=10, is acquired at larger strength of viscoplastic parameter under second-order velocity slip and heat generation with the chemical reaction. In the physical way, the viscoplastic effects of non-Newtonian nanofluid overcome the viscous effects. Increasing phenomena is pretended in the vicinity of channel yε[0,1.286], and while the converse behavior is seen in the vicinity of channel yε[−1.286,0] in the plot of stream function as α rises. Fig. 4(*b*) deals with the effects of Brownian motion (nb) on ψ(y) of nanofluid under heat generation and second-order velocity slips. Here, it is shown that as nb is increase from 0.1 to 2, the graph of ψ(y) is reduced in lower-half of the channel and increased in upper-half of the channel. For small strength of Brownian parameter, the maximum magnitude of stream function is obtained in the region yε[0,1.286], and minimum magnitude is predicted in the region yε[−1.286,0]. But the slope of the stream function is upward within domain yε[0,1.286], and the steep of the stream function is downward in the region yε[−1.286,0]. But the graph is shifted to lower half of the channel as increased to nb=2. Subsequently, it shows that the Brownian motion has a significant act to remove the sinusoidal wave in the parabolic shape and shifting the slope to the downward direction. Additionally, the larger intensity of Brownian motion overcomes the viscoplastic effects. The influence of porosity parameter (Darcy's number (Da)) on ψ(y) of nanofluid is drawn in Fig. 4(*c*) under heat generation and second-order velocity slips. The magnitude of ψ(y) is enhanced in both upper and lower halves by increasing Da from 0.15 to 2. While, under larger porosity effect, say Da=0.1, the wave nature of ψ(y) is strongly affected and a straight line is obtained. Moreover, the larger strength of porous medium overcomes the viscoplastic effect and strongly affected the shape of stream function. Mathematically, it represents a graph of straight line in which ψ(y) is equal to negative value of y−axis. Fig. 4(*d*) represents the variation of ψ(y) at x=π/3 for different values of velocity slip parameters $\left(\beta_{1},\beta_{2}\right)$ under heat generation and porosity effects. Here, the graph of ψ(y) behaves different in each value of $\beta_{1}\;\&\;\beta_{2}$. The magnitude of ψ(y) is larger (smaller) at $\beta_{1}=0\;\&\;\beta_{2}=0.1\;\left(\beta_{1}=0.1\;\&\;\beta_{2}=0\right)$. According to the literature, velocity slip parameters control the stream flow and velocity profile at the boundary walls. Additionally, these parameters reduce the flow resistance from the boundary walls. Because of these physical activities, the stream function is increased immediate the boundary as velocity slips rises. Another important thing is observed, the graphical shape of stream function is also affected at $\beta_{1}=0\;\&\;\beta_{2}=0.1$ and $\beta_{1}=0.1\;\&\;\beta_{2}=0.1$. The effects of radiation parameter (Rn) and Prandtl number (Pr) on ψ(y) of nanofluid is drawn in Fig. 4(*e*) and **(*f*)** under second-order velocity slips and porosity effects. These two figures are plotted under larger porosity effects. The decreasing behavior in ψ(y) is observed in both figures by increasing Rn from 0 to 1 and Pr from 1 to 5. In Fig. (3*e*), it is shown that the larger impacts of conduction heat transfer on the stream function overcome thermal radiation transfer, and in the conclusion, the stream function is declined. While in Fig. (3*g*), as the numeric value of Prandtl number rises the physical effects of kinematic viscosity are enhanced in the stream function, and the influence of thermal diffusivity on the stream function is reduced. Additionally, the substantial intensity of kinematic viscosity has a productive role in decline of stream function under second-order velocity slips effect. The wavy structure of stream function is also distracted because of porosity effects. Fig. 4(*g*) shows the variation of ψ(y) for distinct values of chemical reaction parameter (ξ) under porosity and second-order slip effects. The graph of ψ(y) increased in upper-half and decreased in the lower-half of the geometry by increasing ξ from 0 to 3. Increasing phenomena is noticed in the vicinity of channel yε[0,1.286], and while the converse performance is seen in the vicinity of channel yε[−1.286,0] in the diagram of stream function as ξ rises.

**Fig. 5:**
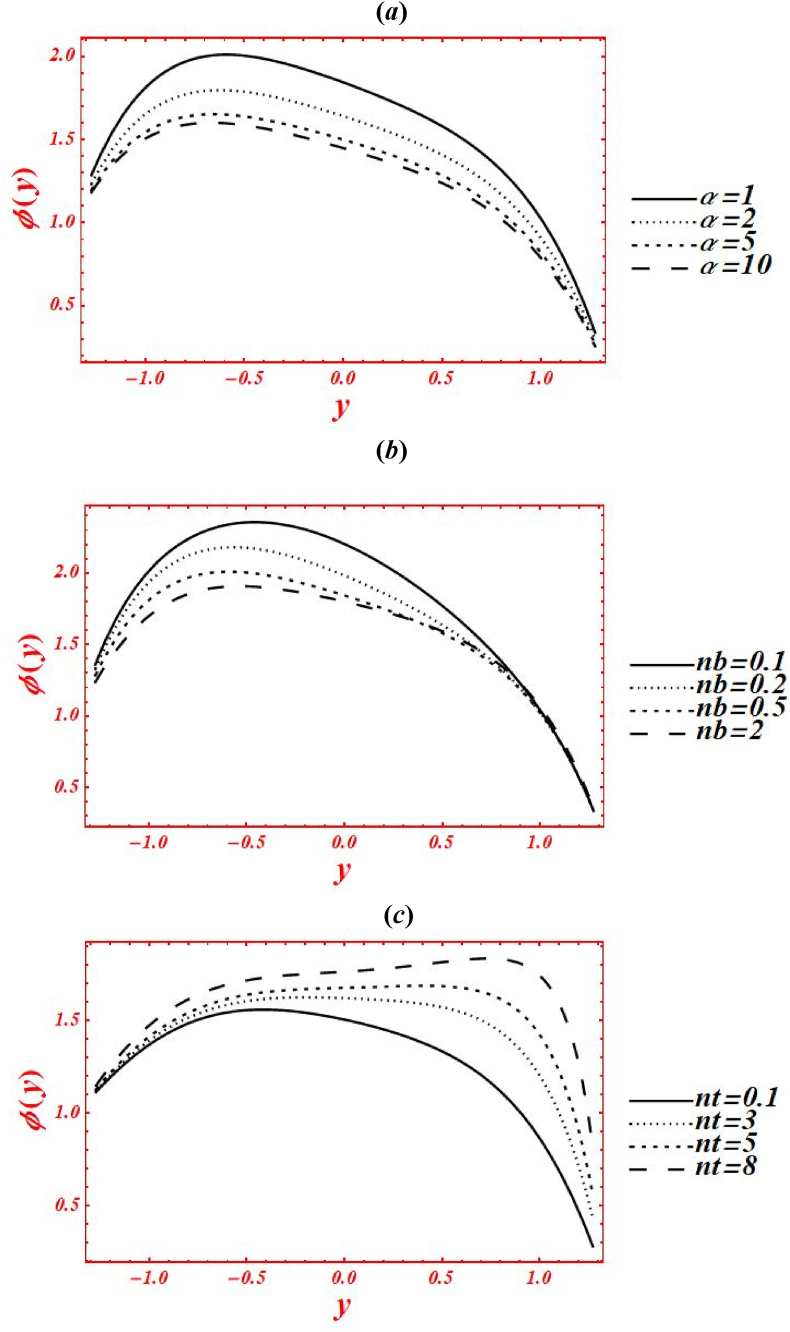

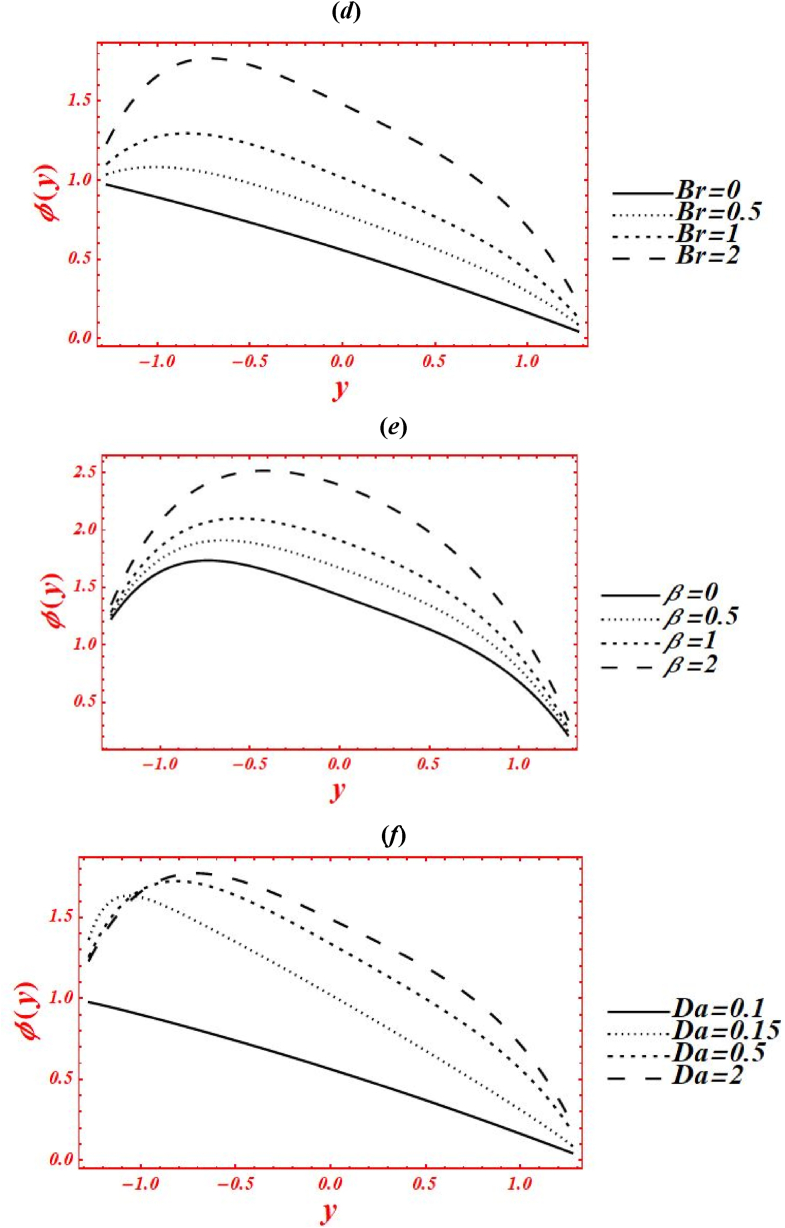

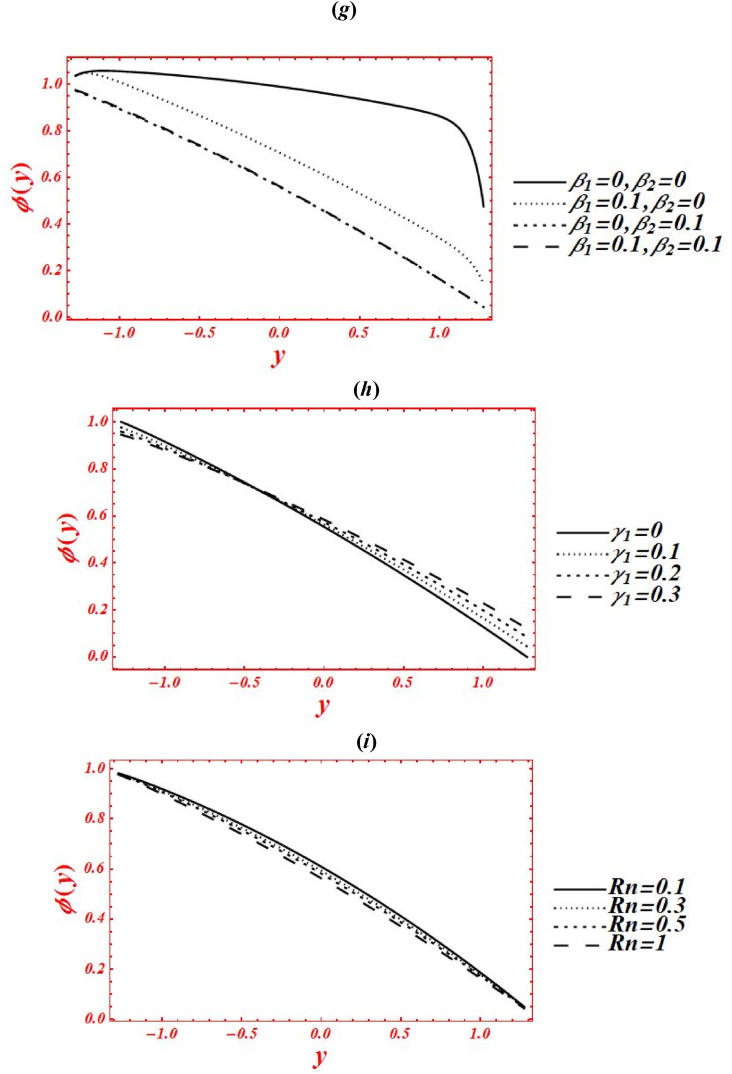

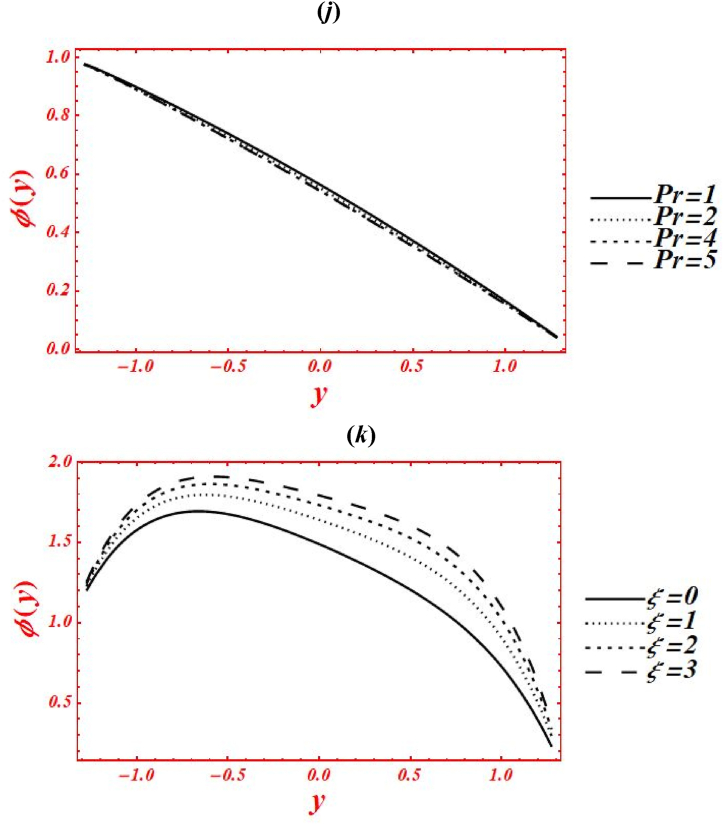
(a–k): (a) Temperature profile for Casson parameter (α), (*b*) Temperature profile for Brownian motion (nb), (*c*) Temperature profile for thermophoresis parameter (nt) , (*d*) Temperature profile for Brinkman number (*Br*), (*e*) Temperature profile for heat source/sink parameter (β), (*f*) Temperature profile for Darcy's number (Da), (*g*) Temperature profile for second-order velocity slips $\left(\beta_{1},\beta_{2}\right)$ (*h*) Temperature profile for thermal slip $\left(\gamma_{1}\right)$, (*i*) Temperature profile for radiation parameter (Rn), (*j*) Temperature profile for Prandtl number (Pr), (*k*) Temperature profile for chemical reaction parameter (ξ).

Fig. 5(*a*–*k*) depict the appearance of flow parameters on the temperature distribution (φ(y)). The effects of Casson parameter (α) on φ(y) under thermal slip, buoyance force and heat generation with the chemical reaction is plotted in Fig. 5(*a*). The magnitude of φ(y) is reduced by increasing Casson (viscoplastic) parameter from 1 to 10. In physical way, it shows that the graph of φ(y) for Casson nanofluid is larger than viscous fluid under thermal slip with the chemical reaction. The larger intensity of viscoplastic parameter overcomes the thermophoresis effect. Fig. 5(*b*) deals with the effects of Brownian motion (nb) on φ(y) of nanofluid under thermal slip. The graph of φ(y) is reduced as nb is increase from 0.1 to 2. Physically, the temperature profile is strongly affected by increasing the collusion and random motion of nanoparticles. The effects of thermophoresis parameter (nt) on φ(y) is plotted in Fig. 5(*c*) under thermal slip and Brownian motion. The magnitude of φ(y) is enhanced by increasing nt from 0.1 to 8. Physically, the larger strength of nt overcomes the Brownian motion and chemical reaction effect. Moreover, the larger intensity of thermophoretic velocity overcomes the temperature gradient and the magnitude of temperature profile is enhanced. Fig. 5(*d*) associated with the impact of Brinkman number (*Br*) on φ(y). The elevation in temperature magnitude is observed by increasing *Br* from 0 to 2. Here, Buoyance force has a dynamic role in enhancement of φ(y). Physical the larger strength of Buoyance force strongly affected the random motion of nanoparticles. Due to this, the augmentation phenomena is noticed in the magnitude of temperature profile. The influence of heat source/sink parameter (β) on φ(y) is drawn in Fig. 5(*e*) under thermal slip and Brownian motion. The enhancement in φ(y) is seen by increasing β from 0 to 2. Subsequently, the thermal conductivity of nanofluid is increased as the heat source rises. Due to larger intensity of thermal conductivity, the drastically enhancement is found in temperature profile. Additionally, the bigger concentration of thermal conductivity on φ(y) overcomes the physical effects of viscoplastic parameter. The effect of porosity parameter (Darcy's number (Da)) on φ(y) is drawn in Fig. 5(*f*) under heat generation and thermal slip. The magnitude of φ(y) is enhanced by increasing Da from 0.1 to 2. Physically, larger strength of porous medium has a vibrant role in the temperature reduction. Besides, the larger strength of porous media overcomes the physical effects of thermophoresis feature. Fig. 5(*g*) and **(*h*)** represent the variation of φ(y) at x=π/3 for different values of velocity slip parameters $\left(\beta_{1},\beta_{2}\right)$ and thermal slip $\left(\gamma_{1}\right)$. The graph of φ(y) is reduced by increasing the second-order velocity slip parameters $\left(\beta_{1},\beta_{2}\right)$ and thermal slip $\left(\gamma_{1}\right)$. In Fig. (*4g*), it is noticed that the shape of temperature profile is strongly affected under velocity slip parameters. Additionally, the larger strength of velocity slip parameters overcome the impacts of heat source/sink parameter and Brinkman number. The physical effects of thermal slip on the temperature profile are noticed under velocity slips effect in Fig. 5(*h*). Additionally, the thermal slip parameter on φ(y) overcomes the heat source/sink effect as $\gamma_{1}$ increases. The effects of radiation parameter (Rn) and Prandtl number (Pr) on the temperature profile is drawn in Fig. 5(*i*) and **(*j*)** under thermal slip and porosity effects. The decreasing behavior in φ(y) is observed in both figures by increasing Rn from 0 to 1 and Pr from 1 to 5. In Fig. (4*i*), it is shown that the larger impacts of conduction heat transfer overcome thermal radiation transfer, and in the conclusion, the temperature profile is declined. Additionally, the abundant intensity of conduction heat transfer strongly affected the shape of temperature profile. While in Fig. (4*h*), as the numeric value of Prandtl number rises the physical effects of kinematic viscosity are enhanced in the temperature profile, and the influence of thermal diffusivity on the temperature profile is reduced. Additionally, the higher intensity of kinematic viscosity has a productive role in decline of temperature distribution. Fig. 5(*k*) shows the variation of φ(y) for distinct values of chemical reaction parameter (ξ) under thermal slip and porosity effects. The graph of φ(y) increased by increasing ξ from 0 to 3. Realistically, the chemical reaction parameter has a drastic act in augmentation of temperature distribution. Additionally, the larger strength of chemical reaction on the temperature distribution overcomes the Brownian motion.

**Fig. 6:**
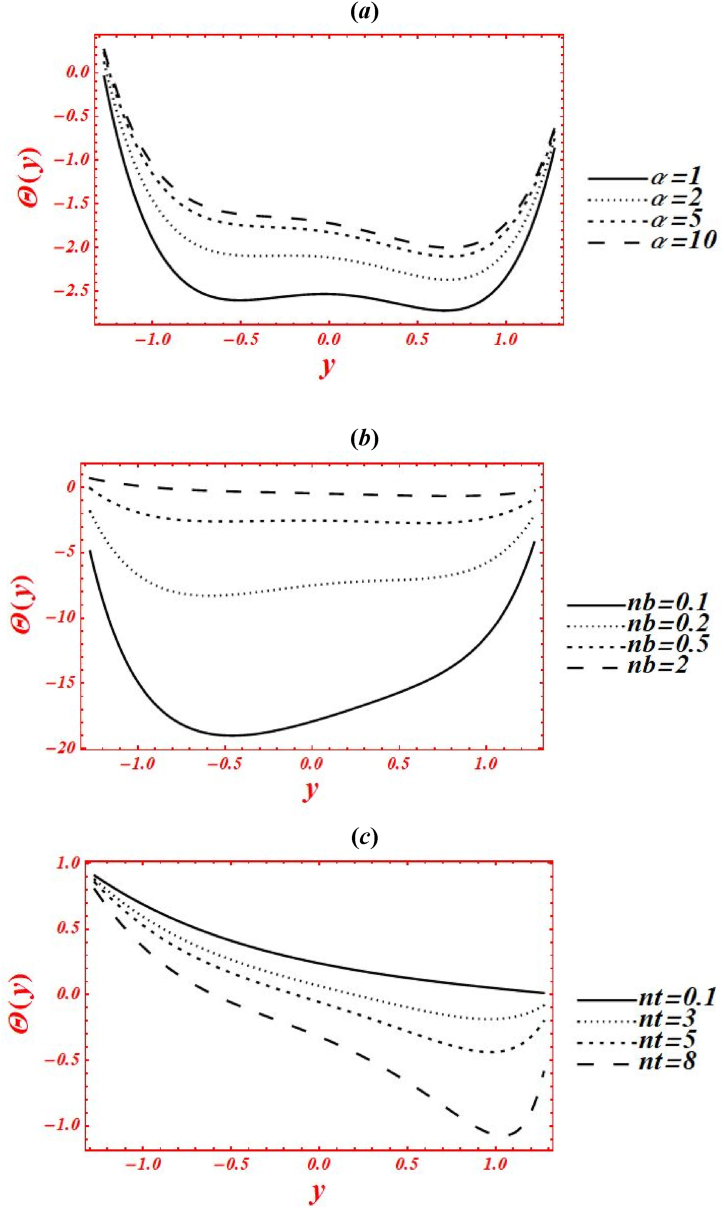

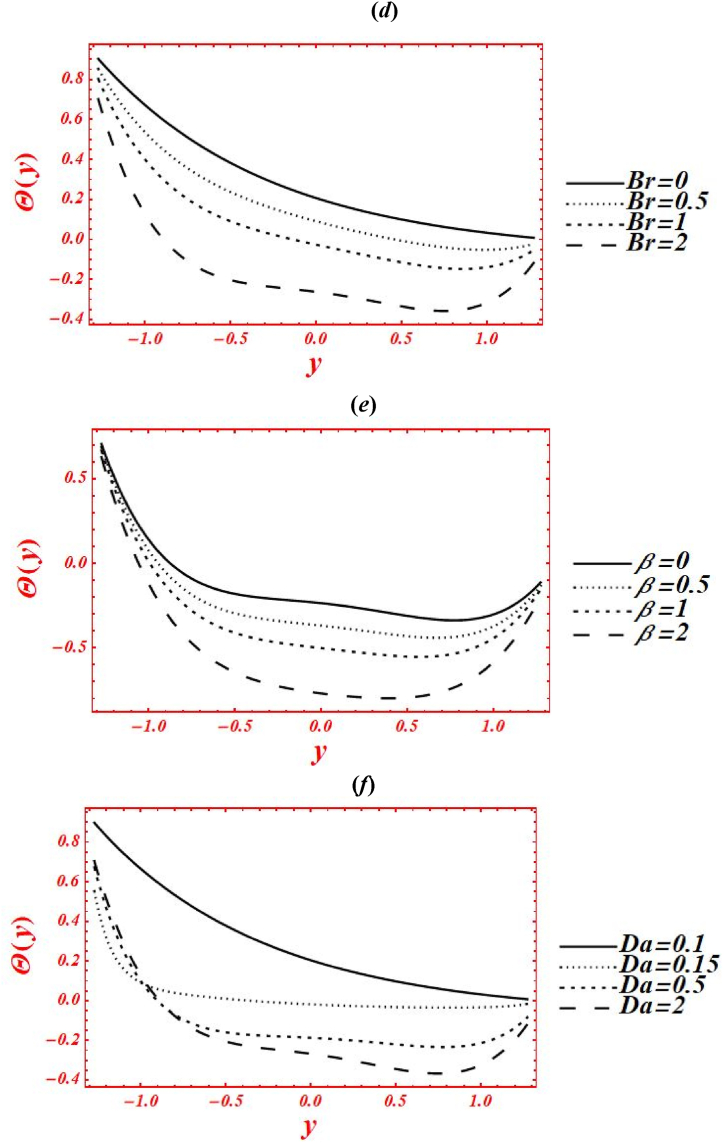

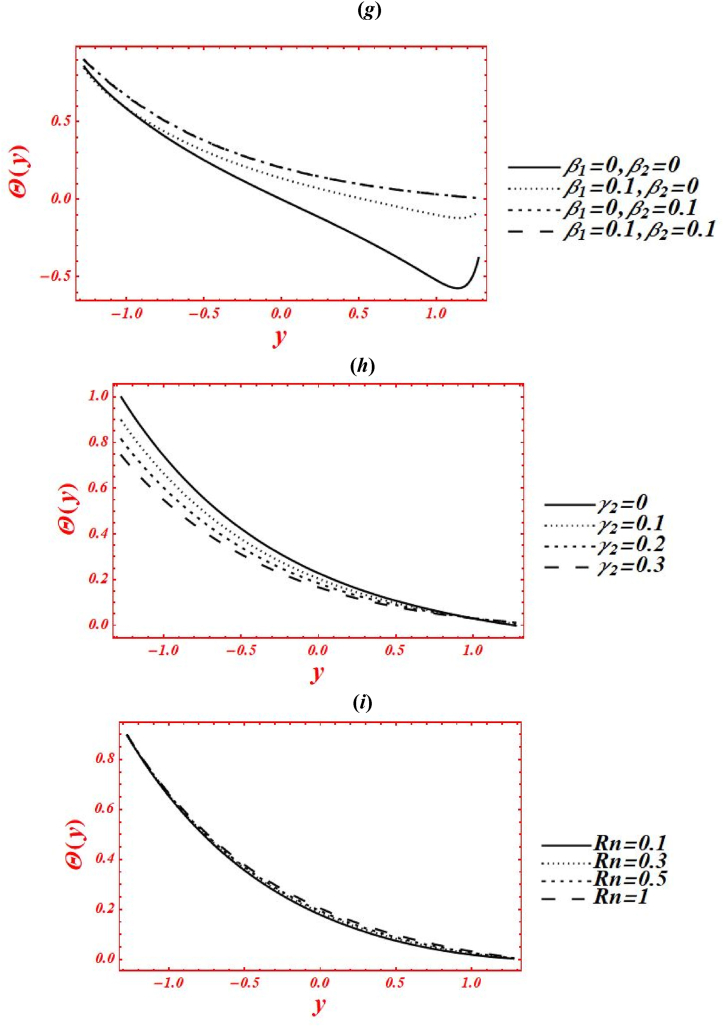

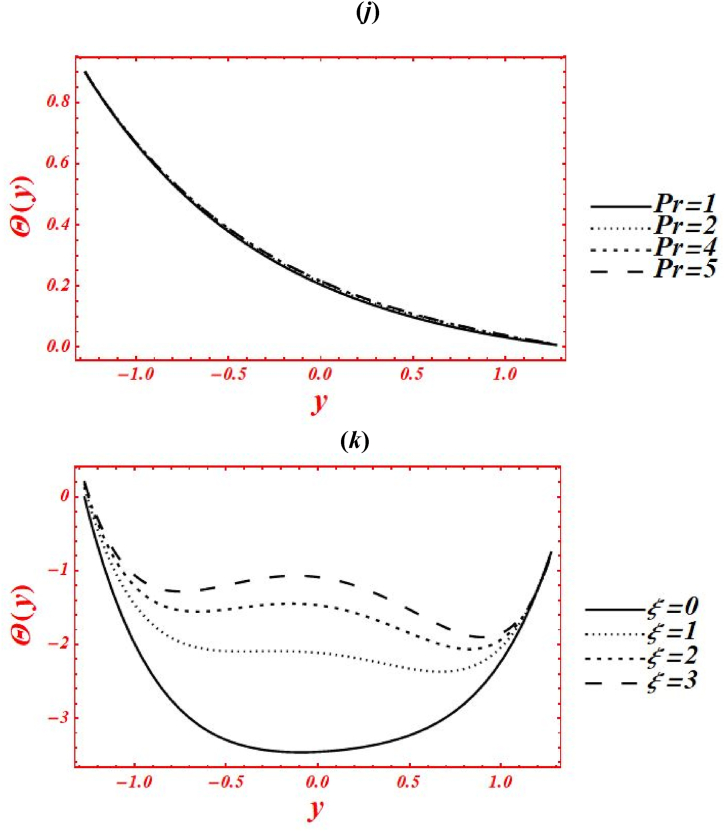
(*a-j*): (a) nanoparticle concentration for Casson parameter (α), (*b*) nanoparticle concentration for Brownian motion (nb), (*c*) nanoparticle concentration for thermophoresis parameter (nt), (*d*) nanoparticle concentration for Brinkman number (*Br*), (*e*) nanoparticle concentration for heat source/sink parameter (β), (*f*) nanoparticle concentration for Darcy's number (Da), (*g*) nanoparticle concentration for second-order velocity slips $\left(\beta_{1},\beta_{2}\right)$, (*h*) nanoparticle concentration for concentration slip $\left(\gamma_{2}\right)$, (*i*) nanoparticle concentration for radiation parameter (Rn), (*j*) nanoparticle concentration for Prandtl number (Pr), (*k*) nanoparticle concentration for chemical reaction parameter (ξ).

Fig. 6(*a*–*j*) depict the influence of embedded parameters on the nanoparticle mass concentration (Θ(y)). All the graphs of nanoparticle concentration showed opposite trend via compared with the graphs of temperature distribution. The effects of Casson parameter (α) on Θ(y) under concentration slip, buoyance force and heat generation with chemical reaction is plotted in Fig. 6(*a*). The magnitude of Θ(y) is reduced by increasing Casson (viscoplastic) parameter from 1 to 10. In physical way, it shows that the graph of Θ(y) for Casson nanofluid is larger than viscous fluid under concentration slips and chemical reaction effect. Additionally, the larger intensity of viscoplastic parameter overcomes the physical effect of heat source/sink parameter and Brinkman number. Fig. 6(*b*) deals with the effects of Brownian motion (nb) on Θ(y) of nanofluid under concentration slip. The graph of Θ(y) is reduced as nb is increase from 0.1 to 2. Whenever the Brownian motion increases, the intermolecular collusion and random motion of nanoparticles are increased. Due to this enhancement, the nanoparticle concentration is boosted. The physical effects of thermophoresis parameter (nt) on Θ(y) is plotted in Fig. 6(*c*) under concentration slip and Brownian motion. The magnitude of Θ(y) is enhanced (reduced) in the right side (left side) of y=0 by increasing nt from 0.1 to 8. Moreover, the larger intensity of thermophoretic velocity overcomes the temperature gradient and the magnitude of nanoparticle concentration is enhanced. Additionally, the graph of nanoparticle concentration is shifted toward the lower half. Fig. 6(*d*) associated with the consequence of Brinkman number (*Br*) on Θ(y). The demotion in magnitude of Θ(y) by increasing *Br* from 0 to 2. Here, Buoyance force has a dynamic role in detraction of Θ(y). Physical the larger strength of Buoyance force strongly affected the random motion of nanoparticles. Due to this, the augmentation phenomena is noticed in the magnitude of nanoparticle concentration. The influence of heat source/sink parameter (β) on Θ(y) is drawn in Fig. 6(*e*) under concentration slip and Brownian motion. The enhancement in Θ(y) is seen by increasing β from 0 to 2. Subsequently, the thermal conductivity of nanofluid is increased on the nanoparticle concentration as the heat source rises. Due to larger intensity of thermal conductivity, the drastically enhancement is found in nanoparticle concentration. Additionally, the larger strength of thermal conductivity on the mass concentration overcomes the physical effects of viscoplastic parameter. The effect of porosity parameter (Darcy's number (Da)) on Θ(y) is drawn in Fig. 6(*f*) under heat generation and mass concentration slip. The magnitude of Θ(y) is decreased as increases Da from 0.1 to 0.15. While, increasing behavior of Θ(y) is seen in the lower half of the channel as rises Da from 0.15 to 2. Farther, the larger strength of porous media on the mass concentration overcomes the physical impacts of thermophoresis feature. Fig. 6(*g*) and **(*h*)** represents the variation of concentration profile at x=π/3 for different values of velocity slip parameters $\left(\beta_{1},\beta_{2}\right)$ and concentration slip $\left(\gamma_{2}\right)$. The graph of Θ(y) is increased by increasing the second-order velocity slip parameters $\left(\beta_{1},\beta_{2}\right)$. While the graph of mass concentration is reduced as increases the concentration slip $\left(\gamma_{2}\right)$. In Fig. 6(*g*), it is noticed that the shape of mass concentration is strongly affected under velocity slip parameters. Additionally, the larger strength of velocity slip parameters on the nanoparticle overcome the physical impacts of heat source/sink parameter and Brinkman number. Additionally, the concentration slip parameter overcomes the heat source/sink effect on the nanoparticle concentration as $\gamma_{1}$ increases. The effects of radiation parameter (Rn) and Prandtl number (Pr) on Θ(y) is drawn in Fig. 6(*i*) & **(*j*)** under concentration slip and porosity effects. The increasing behavior in the mass concentration is observed in both figures by increasing Rn from 0 to 1 and Pr from 1 to 5. In Fig. 6(*i*), it is shown that the larger impacts of conduction heat transfer overcome thermal radiation transfer on the nanoparticle concentration, and in the conclusion, the nanoparticle concentration is declined. Additionally, the abundant intensity of conduction heat transfer strongly affected the shape of the nanoparticle concentration. While in Fig. 6(*h*), as the numeric value of Prandtl number rises the physical effects of kinematic viscosity are enhanced in the nanoparticle concentration, and the influence of thermal diffusivity on the nanoparticle concentration is reduced. Additionally, the larger intensity of kinematic viscosity has a productive role in reduction of the nanoparticle concentration. Fig. 6(*k*) shows the variation of Θ(y) for distinct values of chemical reaction parameter (ξ) under concentration slip and porosity effects. The graph of Θ(y) decreased by increasing ξ from 0 to 3. Realistically, the chemical reaction parameter has a drastic act in reduction of the nanoparticle concentration. Additionally, the larger strength of chemical reaction on the nanoparticle concentration overcomes the Brownian motion.

## 6 Conclusions

Numerical solutions have been derived for non-Newtonian nanofluid rheology via peristaltic pumping in a porous medium under the action of second-order velocity, thermal and concentration slip parameters with a chemical reaction. The long wavelength and creeping theory assumptions, experimental valid for biological flows, have been employed. The numerical simulations, based on the NDSolve technique, have indicated that:

❖ Axial velocity shows decreasing (increasing) behavior in right (left) side of y=0 by increasing viscoplastic parameter, Brownian motion, and chemical reaction parameter. While the radiation parameter and Prandtl number have dynamic role in increment of velocity profile. The Boundary layer is obtained in the graph of axial velocity under larger strength of porous medium.
❖ Stream function shows increasing (decreasing) behavior in right (left) side of y=0 by increasing viscoplastic parameter, Brownian motion, and chemical reaction parameter. While the radiation parameter and Prandtl number have dynamic role in reduction of stream function in both right and left sides of y=0. The wave of stream function is effectively altered under larger strength of porous medium. The Brownian motion parameter shifted the graph of stream function from the upper half to lower half in the vicinity at yε[0,1.286].
❖ The graphs of nanoparticle concentration are opposite to the temperature distribution.
❖ Increasing viscoplastic parameter, Brownian motion, second-order velocity slip parameters, thermal slip, Prandtl number, and radiation parameter strongly decreases the temperature. While, the temperature profile is boosted by increasing the chemical reaction, thermophoresis parameter, Brinkman number, heat source/sink parameter and Darcy number. The graph of temperature distribution is strongly affected under the presence of second-order velocity slip parameters. The magnitude of temperature distribution in the absence of velocity slips is larger in magnitude via related with the presence of velocity slip effects.
❖ Increasing viscoplastic parameter, Brownian motion, concentration slip, and Darcy number strongly decreases the mass concentration. While, the nanoparticle concentration is boosted by increasing the chemical reaction, thermophoresis parameter, Brinkman number, heat source/sink parameter, Prandtl number, radiation parameter and second-order velocity slip parameters. The graph of nanoparticle concentration is strongly affected under the presence of second-order velocity slip parameters. The magnitude of nanoparticle concentration in the absence of velocity slips is larger in the magnitude via compared with the presence of velocity slip effects. Additionally, the graph of nanoparticle concentration is shifted to the upper half in the presence of velocity slips effect.
❖ All the graphs are drawn at fixed value of non-uniform parameter, say φ=0.5.
❖ Some fixed values of rheological parameters are used, i.e, $Gr=0.5,Gm=0.5,Da=1.5,nt=1,nb=0.1,Rn=1,Br=2,\mathrm{Pr}=1,\xi =1,\beta =0.1,\beta_{1}=0.1,\beta_{2}=0.1,\gamma_{1}=0.1,\gamma_{2}=0.1,Q=2,\&\;d=1$**,** to draw the various rheological features.
❖ The consequences of a viscous nanofluid are obtained at α→∞.
❖ The outcomes of a viscous nanofluid without slip effects is obtained at α→∞, $\beta_{1}=0,\beta_{2}=0,\gamma_{1}=0\;\&\;\gamma_{2}=0\text{.}$.
❖ The results of a viscous nanofluid without heat source/sink effect is obtained at →∞ &β=0.
❖ New horizon is developed for researchers and mathematicians to perform the physical aspects of electroosmosis, porous medium, Hall devices, and magnetic device on the nanoliquid transportation in the divergent channel under biological aspects. Additionally, we have ignored the convergent and phase difference parameters impact on the rheological features of current formulation and performed the analysis in the non-uniform channel. Moreover, the different paths of peristaltic waves can be used in the boundary walls of peristaltic pumps and perform dynamic contribution in this horizon.

## Author contribution statement

Walid Aich, Kaouther Ghachem, Muhammad Asad Iqbal: Analyzed and interpreted the data.

Khurram Javid: Conceived and designed the experiments; Performed the experiments.

El Sayed Mohamed Tag-ElDin: Performed the experiments; Contributed reagents, materials, analysis tools or data.

Irfan Ullah: Conceived and designed the experiments; Wrote the paper.

Sami Ullah Khan, Lioua Kolsi: Contributed reagents, materials, analysis tools or data.

## Data availability statement

Data will be made available on request.

## Declaration of competing interest

The authors declare that they have no known competing financial interests or personal relationships that could have appeared to influence the work reported in this paper.

## Nomenclature

$\left(\tilde{X},\tilde{Y}\right)$: Cartesian coordinate (m)
$\left(\tilde{U},\tilde{W}\right)$: velocity components $\left(ms^{-1}\right)$
μ: dynamic viscosity $\left(kgm^{-1}s^{-1}\right)$
t: time $\left(s^{-1}\right)$
P: pressure $\left(m^{2}\right)$
$\mu_{B}$: plastic dynamic viscosity $\left(kgm^{-1}s^{-1}\right)$
σ: wavelength (m)
$\tilde{\varphi }$: temperature (K)
$d_{T}$: thermophoretic diffusion coefficient $\left(m^{2}s^{-1}\right)$
G: gravitational field $\left(m/s^{2}\right)$
*d*_*b*_: Brownian diffusion coefficient $\left(m^{2}s^{-1}\right)$
A: half channel-width
φ: non-uniform (divergent) parameter
Ω: wave speed
$\tilde{\tau }$: Cauchy stress tensor
$\left({\tilde{\tau }}_{\tilde{X}\tilde{X}},{\tilde{\tau }}_{\tilde{X}\tilde{Y}},{\tilde{\tau }}_{\tilde{Y}\tilde{Y}}\right)$: components of extra stress tensor
$\tilde{\Theta }$: nanoparticle phenomena
$\varphi_{p}$: particle density
C: volumetric expansion coefficient
ζ: ratio effective heat capacity of nanoparticles to effective heat capacity of fluid
ς: thermal expansion coefficient
$C_{p}$: volumetric expansion coefficient of nanoparticles
$\varsigma^{\prime }$: expansion with concentration coefficient
$K^{*}$: permittivity of boundary walls
$K_{1}$: chemical reaction parameter
$Q_{0}$: heat source/sink parameter
ϱ: Stefan-Boltzmann constant
k: mean absorption coefficient
φ: dimensionless temperature profile
Θ: dimensionless nanoparticle
Gr: Grashof number
Gm: local nano particle Grashof number
nb: Brownian motion
nt: thermophoresis
$a_{i}$: different amplitudes
$R_{e}$: Reynolds number
δ: wave number
Rn: Brownian motion
Pr: Prandtl number
γ: heat source/sink parameter
Ec: Eckert number
ξ: chemical reaction
Da: Darcy's number (porosity parameter)
Br: Brinkman number
$\tau_{ij}$: extra-stress tensor components
α: viscoplastic parameter
$(\beta_{1}\text{,}$$\beta_{2})$: first and second-order slip parameters
$\gamma_{1}$: thermal slip parameter
$\gamma_{2}$: nanoparticle concentration slip parameter
